# Identification of Novel Thyroid Cancer-Related Genes and Chemicals Using Shortest Path Algorithm

**DOI:** 10.1155/2015/964795

**Published:** 2015-03-22

**Authors:** Yang Jiang, Peiwei Zhang, Li-Peng Li, Yi-Chun He, Ru-jian Gao, Yu-Fei Gao

**Affiliations:** ^1^Department of Surgery, China-Japan Union Hospital of Jilin University, Changchun 130033, China; ^2^The Key Laboratory of Stem Cell Biology, Institute of Health Sciences, Shanghai Institutes for Biological Sciences, Chinese Academy of Sciences, Shanghai 200031, China

## Abstract

Thyroid cancer is a typical endocrine malignancy. In the past three decades, the continued growth of its incidence has made it urgent to design effective treatments to treat this disease. To this end, it is necessary to uncover the mechanism underlying this disease. Identification of thyroid cancer-related genes and chemicals is helpful to understand the mechanism of thyroid cancer. In this study, we generalized some previous methods to discover both disease genes and chemicals. The method was based on shortest path algorithm and applied to discover novel thyroid cancer-related genes and chemicals. The analysis of the final obtained genes and chemicals suggests that some of them are crucial to the formation and development of thyroid cancer. It is indicated that the proposed method is effective for the discovery of novel disease genes and chemicals.

## 1. Introduction

Thyroid cancer (TC) is a typical endocrine malignancy. During the past three decades, its incidence has been nearly tripled in the whole world, such as the United States and other developed countries [1]. Thus, it has been a formidable and urgent task to uncover the mechanism behind it, thereby efficiently improving the medical treatment. Research has been focused on the findings of possible driving genes of this disease, especially those genes with high frequent mutations, over-expressions, or fusions for a long time. Until recent years, this research process just started to accelerate.

With the advent of advanced technology including the next-generation sequencing technologies, findings of genetic and epigenetic alterations are speeding up [2]. In other words, the gradual accumulation of somatic mutations and chromosomal rearrangements that are related to many crucial tumor initiation and development genes has been found [3]. For example, high prevalence of mutations and gene fusions in effectors of the PI3K-AKT and MAPK pathway occurred in most patients with TC, suggesting its important contributions to tumor initiation and development. Meanwhile, dysregulation of hundreds of gene expressions, such as DPP4, MET, LGALS3, and TIMP1, have been common events in this disease [4]. This achievement towards the uncovering of mechanism behind TC is inspiring. However, despite the unprecedented rate of discovery of novel mutations and gene fusions in TC, evidence towards the tumor genesis of TC is still not convincing because of the still large search space.

In addition to the influence of our genomes, it is evident that cancer is also influenced by environmental chemicals from our daily lives. This is partly because environmental exposures can cause DNA mutations and change epigenetic mechanisms [5]. For example, we might contact fluoride and arsenic in drinking water, and toxic gases from burning of fuel and industrial emissions. Current studies show that outdoor air pollution and second-hand smoke often contain chemicals, such as arsenic and polycyclic aromatic hydrocarbons, which further increase risks of numerous cancers [6]. Exposure to toxic level of arsenic can significantly increase DNA methylation of p16 and p53 promoter regions [7] and change miRNA expression [8]. However, many chemicals' effects towards cancer have not been researched and illustrated. Considering the important influences of chemicals towards cancer, we are also interested in searching for novel chemicals related to TC.

We realized that with the simple results from experiments, it would be difficult to meet up our expectation on the detection of novel genes and chemicals related to TC due to the time- and money-consuming process. Thus, more effective and rapid alternative methods must be used to assist the searching process of genes and chemicals related to TC. Considering the efficiency of computational approach, it might be a potential way, which can be used to complete this arduous searching task in a more effective and time-saving way. Until now, several computational methods have been developed in the field of biological network analysis and other related areas, such as construction and analysis of gene regulation, gene coexpression or other biological networks [9–14], and drug designs [15–21]. Recently, some computation methods were proposed to identify new candidate disease genes based on the knowledge of the known disease genes [22–25]. These methods only considered the disease genes. However, it is easy to improve their methods to identify both genes and chemicals that were related to certain disease. In this study, we generalized their methods by constructing a weighted graph containing the information of protein-protein interactions, chemical-chemical interactions, and chemical-protein interactions and applied this method to study TC. Similar to the methods in [22–25], according to known TC-related genes that were collected from TSGene Database [26], UniPort [27], and NCI (National Cancer Institute) [28] and known TC-related chemicals retrieved from CTD (Comparative Toxicogenomics Database) [29], some new candidate genes and chemicals were discovered by our method. The analysis results of these new candidate genes and chemicals showed that some of them are crucial to the formation and development of TC. We hope that this method could contribute to uncovering the mechanism of TC.

## 2. Materials and Methods

### 2.1. Materials

The TC-related genes were collected from three sources: 209 TC-related genes were achieved from UniProt (http://www.uniprot.org/) [27] after we input “human thyroid cancer reviewed” as the keywords; 16 genes were chosen in the catalogue of thyroid cancer from TSGene database (http://bioinfo.mc.vanderbilt.edu/TSGene/search.cgi) [26]; 251 TC-related genes were retrieved from NCI (https://gforge.nci.nih.gov/, released April 2009) [28]. After integrating the above 476 genes, we finally obtained 444 different TC-related genes, which were provided in Online Supporting Information (see S1 in Supplementary Material available online at http://dx.doi.org/10.1155/2015/964795).

The TC-related chemicals were retrieved from CTD (http://ctdbase.org/) [29], which included the interactions between chemicals and genes and their associations with diseases that were manually curated from 110,142 articles (http://ctdbase.org/about/dataStatus.go, accessed 2014 August). Only the 44 chemicals that were markers of TC, were therapeutic to TC, or had known mechanism in TC were analyzed. The pubchem IDs of these chemicals were also provided in Online Supporting Information S1.

### 2.2. A Weighted Graph Constructed from Interactions of Chemicals and Proteins

The core idea of our method is to construct a hybrid weighted graph containing the information of proteins, chemicals, and their associations. This idea has been applied to our previous study on assigning chemicals and enzymes to metabolic pathway [30]. To do that, we employed the information of protein-protein interactions, chemical-chemical interactions, and chemical-protein interactions.

The information concerning protein-protein interaction was retrieved from STRING (Search Tool for the Retrieval of Interacting Genes/Proteins, version 9.1, http://string.embl.de/) [31], a large scale database containing direct (physical) and indirect (functional) interactions of proteins, which are derived from genomic context, high-throughput experiments, (conserved) coexpression, or previous knowledge (refer to http://string.embl.de/). Some computational models have been built based on these information [32–35]. Each obtained interaction contains two proteins and one score, which measures the strength of the interaction, that is, the likelihood of the interaction's occurrence. For latter formulation, let us denote the score of the interaction between proteins *p*_1_ and *p*_2_ by *S*_*pp*_(*p*_1_, *p*_2_). In particular, if proteins *p*_1_ and *p*_2_ cannot comprise an interaction according to the current data in STRING, *S*_*pp*_(*p*_1_, *p*_2_) was set to zero.

The information concerning chemical-chemical interaction and chemical-protein interaction was retrieved from STITCH (Search tool for interactions of chemicals, version 4.0, http://stitch.embl.de/) [36], a sister project of STRING which provides the known and predicted interactions of chemicals and proteins. These interactions are confirmed by evidence derived from experiments, databases, and the literature (refer to http://stitch.embl.de/). Each obtained chemical-chemical interaction contains two chemicals and five scores: “Similarity,” “Experiment,” “Database,” “Textmining,” and “Combined_score,” which measure the strength of the interaction from different aspects, such as their structures, activities, reactions, cooccurrence in literature, and integration of the above information. To widely indicate the interaction between chemicals, we used the last score, that is, “Combined_score,” to measure the strength of the interaction. For two chemicals *c*_1_ and *c*_2_, the “Combined_score” of the interaction between them was denoted by *S*_*cc*_(*c*_1_, *c*_2_). Similarly, *S*_*cc*_(*c*_1_, *c*_2_) was set to zero if *c*_1_ and *c*_2_ do not occur as an interaction in STITCH. Each obtained chemical-protein interaction contains one chemical, one protein, and five scores. With the similar argument, we used the “Combined_score” to indicate the strength of the interaction between one chemical and one protein. Let *S*_*cp*_(*c*, *p*) denote the “Combined_score” of the interaction between chemical *c* and protein *p*. Also, *S*_*cp*_(*c*, *p*) = 0 if *c* and *p* cannot comprise a chemical-protein interaction. It is necessary to point out that all chemicals in the retrieved chemical-chemical and chemical-protein interactions must have records in KEGG (Kyoto Encyclopedia of Genes and Genomes) [37] because the number of chemicals in STITCH is too large and most of chemicals have few associations with human tissues.

Based on the information concerning protein-protein interactions, chemical-chemical interactions, and chemical-protein interactions, a weighted graph *G* = (*V*, *E*) was constructed as follows: *V* contained all proteins and chemicals occurring in the above three kinds of information and *E* consisted of all pairs of nodes such that the corresponding proteins or chemicals can comprise an interaction. It is easy to know that each edge in *G* represented an interaction. On the other hand, as mentioned in the above paragraph, each interaction was assigned a score to indicate its strength; that is, different interactions may have different strength. To note this fact in *G* and use the shortest path algorithm to search for new candidate genes and chemicals, each edge was labeled a weight as follows. Since the range of the interaction scores is between 1 and 999, the weight of an edge *e* with endpoints *n*_1_ and *n*_2_ was defined by

(1) w(e)=1000−Spp(p1,p2)  If  n1  and  n2  represented  proteins  p1  and  p21000−Scp(c,p)  If  n1  and  n2  represented  chemical  c  and  protein  p1000−Scc(c1,c2)  If  n1  and  n2  represented  chemicals  c1  and  c2.

**Figure 1:**
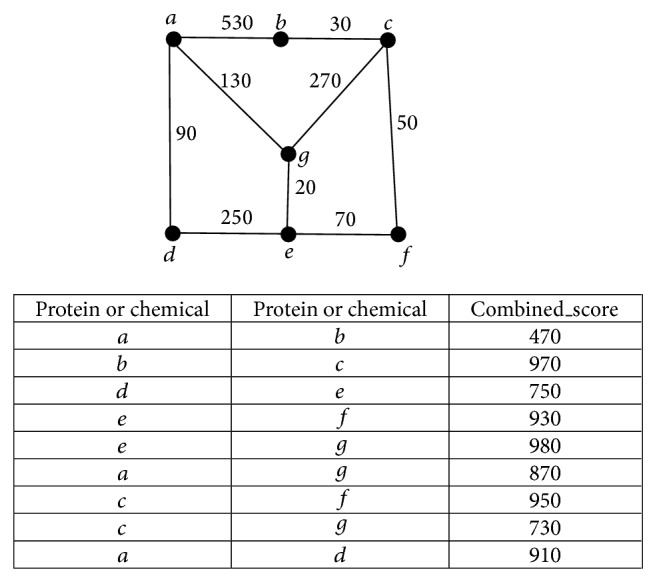
An example to display the construction of the weighted graph, where *a*, *b*, and *c* represent chemicals and *d*, *e*, *f*, and *g* represent proteins.

To clearly display the procedures of construction of the graph, a small example is shown in Figure 1. In the example, there are three chemicals *a*, *b*, and *c* and four proteins *d*, *e*, *f*, and *g*. The interactions, including their “Combined_score,” between them are listed in the table of Figure 1 and the constructed graph based on these interactions is shown at the top of Figure 1.

### 2.3. Method for Discovery of New Candidate Genes and Chemicals

The following method for finding new candidate TC-related genes and chemicals was almost same as that in our previous study [25]. The only difference was that the input of the current method contained both genes and chemicals, while the method in [25] only considered genes. Readers can refer to our previous study [25] for the detailed procedures of the method and its principle. For the integrity of this study, the brief description of the method was as follows: (I) search all shortest paths connecting any pair of TC-related genes and chemicals using Dijkstra's algorithm [38]; (II) for each node (gene or chemical) in *G*, count its betweenness that was defined as the number of paths containing it as an inner node; (III) select the nodes (genes or chemicals) with betweenness larger than zero as candidate genes and chemicals; (IV) produce 1,000 sets by randomly selecting nodes (genes or chemicals) from *G*; the numbers of genes and chemicals in each set were the same as those in known TC-related gene and chemical set; (V) for each set, search all shortest paths connecting any pair of genes or chemicals in *G*; (VI) count the betweenness of candidate genes and chemicals on each randomly produced sets; (VII) for each candidate gene and chemical, compare its betweenness on known TC-related gene and chemical set and those on randomly produced sets, thereby calculating its permutation FDR that was defined as “the number of randomly produced sets on which the betweenness was larger than that on the known TC-related gene and chemical set”/1000.

## 3. Results and Discussions

### 3.1. Candidate Genes and Chemicals

Of the 444 TC-related genes and 44 TC-related chemicals, we searched the shortest paths in *G* such that the endpoints of them were TC-related genes or TC-related chemicals. Accordingly, the betweenness of each gene and chemical in *G* was computed, obtaining 636 candidate genes and 174 candidate chemicals whose betweenness was larger than zero; that is, these genes and chemicals occurred in at least one shortest path as inner nodes. These genes and chemicals are listed in Online Supporting Information S2, in which their betweenness is also provided.

To exclude false discoveries, the permutation test was executed by constructing 1,000 randomly selected gene and chemical sets for calculating the permutation FDR of each candidate gene and chemical, which is also provided in Online Supporting Information S2. Then, we selected 0.05 as a threshold to exclude false discoveries, obtaining 169 candidate genes and 49 candidate chemicals with permutation FDRs smaller than 0.05. The information of these genes and chemicals is available in Online Supporting Information S3. For convenience, we termed these genes and chemicals as significant candidate genes and significant candidate chemicals, respectively. The following discussion was based on these significant candidate genes and significant candidate chemicals.

### 3.2. Gene Enrichment Analysis

DAVID [39] is a powerful tool that could be used to make integrative and systematic of large gene lists. Thus, it was used in this study to analyze the 169 significant candidate genes. The analysis results included two parts: KEGG pathway enrichments (Online Supporting Information S4) and gene ontology (GO) enrichments (Online Supporting Information S5). GO enrichments include three parts: biological process enrichment (BP enrichment), cellular component enrichment (CC enrichment), and molecular function enrichment (MF enrichment). Since our method was mainly based on protein-protein interactions, BP enrichment analysis was more convincing, while other two results were not very reasonable. Thus, we only gave the discussion based on the BP enrichment.

**Figure 2:**
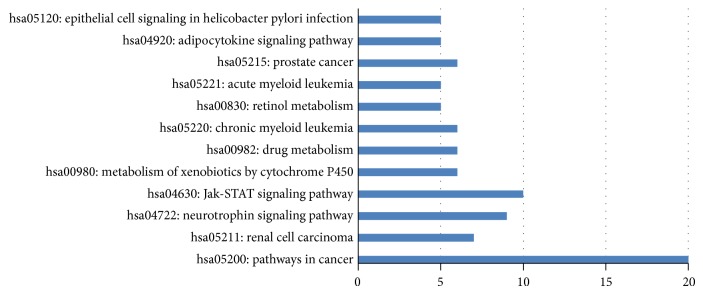
The top twelve KEGG pathways that were enriched by 169 significant candidate genes.

For the KEGG pathway enrichment analysis results, 169 candidate genes are enriched in 19 KEGG pathways (see Online Supporting Information S4). Among these 19 KEGG pathways, twelve of them were with *P* value (modified Fisher exact *P* value) less than 0.05.  Figure 2 shows these twelve KEGG pathways and the number of enriched genes among the significant candidate genes (“count”). Hsa05200 (pathways in cancer, “count” = 20) is the most significant pathway, which enriched 20 significant candidate genes, such as FGFR2, FGF6, DVL3, EPAS1, and PPARG. Since all these genes enriched in this pathway were reported related to cancer formation and development, it further revealed the validity of our method. Hsa05211 (renal cell carcinoma, “count” = 7) is the second significant pathway with 7 genes related to renal cell carcinoma. Hsa04722 (neurotrophin signaling pathway, “count” = 7) is the third significant pathway, enriching 7 genes, such as KRAS, PLCG1, and NTF3. Among them, NTF3 in neurotrophin signaling pathway has been reported with the association to cancer [40]. Other pathways, such as hsa05221 (acute myeloid leukemia, “count” = 5) and hsa05215 (prostate cancer, “count” = 6), also revealed that the significant candidate genes are associated with cancer.

**Figure 3:**
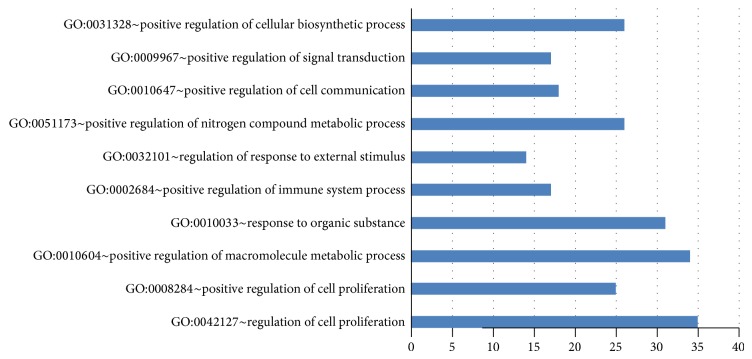
The top ten GO terms that were enriched by 169 significant candidate genes.

For the BP enrichment analysis, results are shown in Online Supporting Information S5. Ranked by *P* value, top ten BP GO terms are depicted in Figure 3. The mainly enriched GO terms are associated with cell proliferation. For example, genes in GO:0042127 (regulation of cell proliferation, “count” = 35) and GO:0008284 (positive regulation of cell proliferation, “count” = 25) are all reported related to cell proliferation. Also, GO:0010604 (positive regulation of macromolecule metabolic process, “count” = 34) and GO:0051173 (positive regulation of nitrogen compound metabolic process, “count” = 26) are associated with metabolic process. Since proliferative signaling and activating metastasis are two hallmarks of cancer [41], it is convincing that the result of BP enrichment analysis further supports the validity of our method.

Thus, this enrichment analysis further proved the importance of genes discovered by our method. We hope that it could be used to gain better understandings of the mechanism of TC.

### 3.3. Analysis of Some Significant Candidate Genes

Among 169 significant candidate genes, we selected some important genes to elucidate their potential values to be TC-related genes. Since they have been reported to be associated with the tumorigenesis or development of other types of cancers, we thought it might lend credence to our method and make our findings more convincing.

The gene CYP2B6 (cytochrome P450, family 2, subfamily B, polypeptide 6) mainly encodes enzymes which are involved in many reactions, specifically in anticancer drug metabolism. A report based on one Japanese population showed that polymorphism of CYP2B6 is significantly associated with prostate cancer risk [42]. Also, decreased expression of CYP2B6 is shown in prostate cancer, and it has been recognized as growth inhibitory [43].

FURIN, also known as PACE, encodes furin protein. High expression of furin has been detected in different cancer types, such as ovarian cancer [44] and head and neck cancer cells [45]. And the inhibition of its expression can help decrease the tumorigenesis of cancers [46]. Also, furin overexpression can promote cell invasion in human hepatoma cell lines, which plays a role in the development of hepatocellular carcinoma [47]. Moreover, the gene may involve in the activity of Notch, and the Notch pathway is important during the medullary thyroid cancer (MTC) [48].

MERTK (c-mer proto-oncogene tyrosine kinase) is a protein-coding gene, which belongs to the MER/AXL/TYRO3 receptor kinase family and encodes cell-surface transmembrane receptors that contain regulated kinase activity [49]. Research has found that MERTK is overexpressed in a variety of cancers, such as prostate cancer, non-small-cell lung cancer, and breast cancer [50]. Also, its overexpression can result in the activation of oncogenic signaling pathways and drive cell transformation in cancer cells [51].

OAS2 (2′-5′-oligoadenylate synthetase 2, 69/71 kDa) is involved in immune response of viral infection, because it activates RNase L as a result of the elimination of viruses. In a recent study of cervical cancer, researchers found that genes related to antiviral response were increasingly expressed, including OAS2 which is directly involved in viral RNA degradation [52].

PPARG (peroxisome proliferator-activated receptor gamma) is a member of PPAR subfamily of nuclear receptors, which plays a crucial role in the regulation of gene transcription and adipocyte differentiation. Currently, the activation of PPARG has been recognized as one key step in colorectal cancer progression [53], and its deacetylation can determine lipid synthesis and growth in breast tumor [54].

To summarize, even though these 169 significant candidate genes have not been found associated with TC until now, a wealth of evidence has proved their relations to other types of cancer. Therefore, previous researches have validated the reliability of our method and the importance of our findings. We hope our method will be helpful to search novel TC-related genes and be further promoted to the exploration of other biological questions.

### 3.4. Analysis of Some Candidate Chemicals

Besides the significant candidate genes, we also discovered 49 significant candidate chemicals that are deemed to be related to thyroid cancer development. Most of them (29 out of 49) can be supported by published literatures. Here, we only gave detailed discussions for three of them. All of these 29 chemicals are briefly discussed in Online Supporting Information S6.

Chloride ion (CID000000312) is a common ion in human cells, which plays a crucial role in cell invasion due to its ability to change the osmotic balance between the inner- and extra-cellular space [55]. The reason behind invading cancer cells that can pass though extracellular matrix is partly because it has the ability to reduce its volume. Several major chloride channels on the cell membrane are responsible for this invasive behavior of cancer cells. Research has found that inhibition of the sodium-potassium-chloride cotransporter isoform-1 (NKCC1) can decrease cell invasion by 50% [56].

Hydrogen cyanide (HCN, CID000000768) is the product of various tobaccos, existing in the smoke as a colorless gas. In the study of gastroesophageal cancer based on selected ion flow tube mass spectrometry (SIFT-MS), hydrogen cyanide is significantly different between cancer and healthy groups [57]. Hydrogen cyanide is also recognized to have cardiovascular and respiratory toxicity, which might be a potential factor to cause lung cancer [58].

Aniline (CID000006115) consists of a phenyl group attached to an amino group, and it is the precursor of industrial chemicals. It is reported that the incidence of bladder cancer is clearly related to exposure to aniline [59]. Potential reasons might be due to an increase in iron overload in the spleen and upregulation of TNF-*α*, IL-1, and IL-6. Also, the expression of cyclin dependent kinases (CDKs) is upregulated by aniline [60].

## 4. Conclusion

During the fight with thyroid cancer, discovery of its related genes and chemicals and uncovering the mechanism behind it are important to today's research and future's drug design for designing effective treatments. Only with the assistance of experiment methods would be an onerous and low efficient way. In this study, we sufficiently used known resource, such as protein-protein interactions, chemical-chemical interactions, chemical-protein interactions, and known thyroid cancer-related genes and chemicals, to search new candidate thyroid cancer-related genes and chemicals by the shortest path algorithm. The proposed method generalized our previous method that can only discover disease genes. Further analysis of the selected genes and chemicals implies that some of them have direct or indirect relationship with the formation and development of thyroid cancer, thereby suggesting the effectiveness of our method. We hope that our method and the findings could shed new light on the mechanism research of thyroid cancer.

## Supplementary Material

The Supplementary Material contains six files. In detail, Online Supporting Information S1 lists thyroid cancer-related genes and chemicals; Online Supporting Information S2 lists 636 candidate genes and 174 candidate chemicals; Online Supporting Information S3 lists 169 significant candidate genes and 49 significant candidate chemicals; Online Supporting Information S4 lists KEGG enrichment results of 169 significant candidate genes; Online Supporting Information S5 lists GO enrichment results of 169 significant candidate genes; Online Supporting Information S6 lists the discussion of 29 significant candidate chemicals.
