# Supplementary material for: Identification of Novel Thyroid Cancer-Related Genes and Chemicals Using Shortest Path Algorithm
Source: Biomed Res Int. 2015 Mar 22;2015:964795. doi: 10.1155/2015/964795 (PMC4385622; doi:10.1155/2015/964795)
Supplement: Supplementary file 1 — The Supplementary Material contains six files. In detail, Online Supporting Information S1 lists thyroid cancer-related genes and chemicals; Online Supporting Information S2 lists 636 candidate genes and 174 candidate chemicals; Online Supporting Information S3 lists 169 significant candidate genes and 49 significant candidate chemicals; Online Supporting Information S4 lists KEGG enrichment results of 169 significant candidate genes; Online Supporting Information S5 lists GO enrichment results of 169 significant candidate genes; Online Supporting Information S6 lists the discussion of 29 significant candidate chemicals. [file 964795.f1.zip › Supp-S1.pdf]

Online Supporting Information S1. 444 thyroid cancer-related genes retrieved from Uniprot, TSGene database, NCI and 44 thyroid cancer-related chemicals obtained from CTD.

(1) Thyroid cancer-related genes

SLC3A2
ABCA3
ABCB10
AFAP1L2
AGR2
AKAP13
AKT1
ADPRHL2
BCAS3
BMP2K
CALCOCO1
CDH1
CTSH
CPQ
CCDC6
CDK13
CDKN1B
ITGB3BP
C8orf4
TRIP10
CLUAP1
CNIH1
CNTN4
C16orf58
CREB1
CTNNBL1
CST4
DCUN1D1
DDR1
DHRS3
SDHB
SDHD
DICER1
DUSP23
DUSP26
EAF2
ELP3
ENO1
EP300

FAM110B
FAM83A
FCRL3
FGF10
FGF2
FGF8
FHIT
FLNB
FLNC
GAL3ST4
GADD45GIP1
GATA3
GDPD5
GALNT10
GALNT12
GNAS
GOLGA5
AGPAT9
GPR56
HR
HEMGN
HPN
HID1
HIPK2
HSPB1
HOXA3
INSR
ISLR
ITPA
JMJD1C
KAT6B
KIDINS220
KDM8
KLLN
KISS1
KISS1R
KIT
CAMKK2
KLK14
KLK15
KLK6
KLK7
PKM
RPS6KA1

RPS6KA2
LAMP3
SLC7A5
LDOC1
LGR4
LRP1B
LRP2
LHCGR
MTDH
LZTFL1
MAP3K8
MARK4
MED14
MET
MLH1
MOB1A
MOB1B
ABCC2
MTUS1
MUC1
CD164
MUC4
MYH14
MYOT
NAA15
NCOA2
NCOA1
NCOA3
NCOA4
NCOA6
NDUFA13
FAM129A
NOL7
NSD1
NTRK1
NUCKS1
NUPR1
KIAA0101
TRIP13
PCM1
MPO
PGAP3
PI15
PIK3CA

PLAGL1
PLK1
PLK2
PKMYT1
PROM2
PSMC5
PRUNE2
PRUNE
PSMB6
PSME3
PTEN
PTK7
PTPRJ
RARB
HRAS
ERC1
RET
RILP
RPL6
RNF135
RNF139
RNF213
RNF14
RREB1
RXFP2
S100A8
S100A9
SLC35F6
SAA1
SASH1
SLC5A8
SCN9A
SEP15
SCGB1D1
SCGB2A1
SIGMAR1
SHH
ST6GALNAC6
SIRT7
SMAD3
SORL1
SORT1
SPDYA
SRA1

SSX1
SSX2
STK33
SYDE2
TMEM184C
TP53INP2
TACC2
TBX1
TNS3
TFG
THRA
TRIM24
TINAGL1
TMEM205
TMEM234
TNFSF11
TNFSF13
TOPORS
TPM3
TPR
TNFRSF11B
THRAP3
TRERF1
TRIM27
TRIM33
TRIB1
TRIP4
TRIP12
TSHB
TSHR
TTBK1
TTR
TTLL3
UBE2V1
AXL
WNT4
ZNF197
ZNF331
THRB
CDKN2A
WWOX
TP73
CDC73
RASSF2

DAPK1
ERRFI1
TIMP3
PRKAR1A
RASSF10
MST1
ADPRH
NOTCH1
UCHL1
SSTR2
FLT1
IL10
RAB23
PAM
E2F3
INSL3
GPT
S100A6
IL6R
SERPINA1
IFI16
DIO1
JUND
TSG101
SKP2
DAO
MCM7
NTRK2
BRCA1
CCK
PAX8
RASSF1
GSTT1
VDR
FGFR1
CDC25B
PGP
GHRH
BRCA2
ABCG2
WNT5A
XRCC1
CYP1A1
CD82

LSM7
SSTR3
BRD4
CREM
FGD3
SOCS1
NNMT
SSTR5
NMTC1
COX1
JUNB
CRH
PRLR
NF1
DCN
HMGA1
EIF4E
GAS6
PDK1
IL6
CITED1
TIMP1
CD68
CASR
DPP4
CTNNB1
PDE4A
GFRA1
CD40
NRAS
VIP
GSTP1
E2F4
TTK
CDC25A
GFRA3
ID1
WRN
S100A2
FGFR3
IL8
COX2
BAX
GPC3

AES
FRS2
DDC
FUT8
SYNGR2
EGF
CDKN1A
MT3
DUOX2
S100A4
TPO
DOK1
MIR187
HK2
GFRA2
GRK5
FLT3
CDX2
BUB1
STAT3
TCEAL4
CD97
GAPDH
CAPN5
TIMM44
CD34
MICA
RLN2
VGF
MMP11
TTF1
TTF2
APOB
BCL2
WT1
TERT
CD36
RPH3AL
AR
FAS
MINPP1
NTRK3
PRL
HGF

CD44
PGR
EPO
MGMT
MCM5
PTH
DCK
AFP
MMP9
RXRG
NDRG1
SRC
VCP
MIR146A
RBP3
CD63
MIR222
LIF
SHBG
CRYAB
TIMP2
S100B
SLC26A4
CDK4
TK1
QPCT
BRAF
MMP1
GSTO1
SPP1
MT1G
CRABP1
TGFA
SFTPB
SSTR1
GAB1
DIO2
TLR3
FRA16D
TRH
FCGBP
SERP1
HDAC1
ERBB2

MNG1
DUOX1
AKAP9
STC1
CD5
BMP7
C1D
TNF
EGFR
TCO
E2F1
APC
CYP3A5
IGFBP1
ZNF20
VHL
ETS1
CSNK1G2
MDM2
CXCR4
LPA
ID3
CALCA
SST
PCNA
MEN1
IL1B
TFF1
NPY
CIITA
TFF3
CYP2D6
MCL1
CDK2
SMAD7
EGR1
IL2
PIN1
FGFR4
MIR146B
AHR
MMP2
FN1
CCL2

GDNF
IGF1
EPOR
G6PD
ECM1
GSTM1
ICAM1
CD55
RB1

(2) Thyroid cancer-related chemicals
CID000000264
CID000000807
CID000001322
CID000002078
CID000003213
CID000003715
CID000004616
CID000004763
CID000005234
CID000005323
CID000005327
CID000005569
CID000005921
CID000005978
CID000007416
CID000007567
CID000007577
CID000007579
CID000008765
CID000011164
CID000011975
CID000012699
CID000018692
CID000023978
CID000031369
CID000031404
CID000031703
CID000039385
CID000053462
CID000053665
CID000064139
CID000064142
CID000091480

CID000441140
CID000444795
CID000445154
CID000657298
CID001349907
CID002723650
CID002723790
CID003034368
CID005353562
CID009562060
CID023673461
