# Supplementary material for: Identification of Novel Thyroid Cancer-Related Genes and Chemicals Using Shortest Path Algorithm
Source: Biomed Res Int. 2015 Mar 22;2015:964795. doi: 10.1155/2015/964795 (PMC4385622; doi:10.1155/2015/964795)
Supplement: Supplementary file 1 — The Supplementary Material contains six files. In detail, Online Supporting Information S1 lists thyroid cancer-related genes and chemicals; Online Supporting Information S2 lists 636 candidate genes and 174 candidate chemicals; Online Supporting Information S3 lists 169 significant candidate genes and 49 significant candidate chemicals; Online Supporting Information S4 lists KEGG enrichment results of 169 significant candidate genes; Online Supporting Information S5 lists GO enrichment results of 169 significant candidate genes; Online Supporting Information S6 lists the discussion of 29 significant candidate chemicals. [file 964795.f1.zip › Supp-S2.pdf]

Online Supporting Information S2. 636 candidate genes and 174 candidate chemicals with betweenness larger than zero and their permutation FDRs

Gene or chemical ID	Gene or chemical name	Betweenness	Permutation FDR
ENSP00000206249	ESR1	21841	0
ENSP00000226574	NFKB1	963	0
ENSP00000227507	CCND1	6321	0
ENSP00000259526	NOV	482	0
ENSP00000263851	IL7	482	0
ENSP00000268655	ZNF174	1	0
ENSP00000269138	SS18	963	0
ENSP00000295108	NEUROD1	482	0
ENSP00000301012	MVD	482	0
ENSP00000302640	UBTF	482	0
ENSP00000302648	NRTN	482	0
ENSP00000304811	UGT2B7	963	0
ENSP00000306157	IL7R	482	0
ENSP00000327213	NRIP1	963	0
ENSP00000334181	KDM4D	482	0
ENSP00000337915	CYP3A4	4122	0
ENSP00000349508	CHD4	482	0
ENSP00000360973	AGTR2	482	0
ENSP00000368066	HAO1	482	0

ENSP00000368981	SLC35B3	482	0
ENSP00000377941	ACTN1	962	0
ENSP00000262809	ELL	482	0.001
ENSP00000270349	SLC6A3	956	0.001
ENSP00000295408	MERTK	962	0.001
ENSP00000338207	LMO1	482	0.001
ENSP00000346015	RPL27A	477	0.001
ENSP00000348089	ERCC6	482	0.001
ENSP00000357255	BGLAP	482	0.001
ENSP00000368754	BAZ2A	482	0.001
ENSP00000369396	SLC4A11	482	0.001
ENSP00000416387	FBLIM1	43	0.001
ENSP00000011684	PLEKHG6	482	0.002
ENSP00000262367	CREBBP	5560	0.002
ENSP00000276297	DLC1	482	0.002
ENSP00000327246	VIPR1	476	0.002
ENSP00000331358	GAST	997	0.002
ENSP00000335544	CCKBR	696	0.002
ENSP00000351605	FZD6	482	0.002
ENSP00000362690	NR5A1	482	0.002
ENSP00000371532	VLDLR	494	0.002
ENSP00000380514	HAGH	3	0.002

ENSP00000391998	ARTN	482	0.002
ENSP00000397297	NTF3	484	0.002
ENSP00000274565	SPINK7	482	0.003
ENSP00000290330	SNF8	482	0.003
ENSP00000370748	IDI1	482	0.003
ENSP00000417763	NAA10	482	0.003
ENSP00000253339	LATS1	482	0.004
ENSP00000331288	TMEM173	482	0.004
ENSP00000269485	TNFRSF11A	962	0.005
ENSP00000284818	LY96	962	0.005
ENSP00000323328	SIGLEC7	478	0.005
ENSP00000339151	IKBKB	501	0.005
ENSP00000344741	INSIG1	482	0.005
ENSP00000244007	PLCG1	2851	0.006
ENSP00000263208	HIRA	482	0.006
ENSP00000304169	PITX2	482	0.006
ENSP00000380033	DDX17	482	0.006
ENSP00000410294	FGFR2	964	0.006
ENSP00000227163	SPI1	482	0.007
ENSP00000279247	CAPN1	470	0.007
ENSP00000322142	ING5	482	0.007
ENSP00000401303	SHC1	2222	0.007

ENSP00000241052	CAT	483	0.008
ENSP00000245157	BBS2	954	0.008
ENSP00000346566	CKAP5	482	0.008
ENSP00000249373	SMO	482	0.009
ENSP00000250003	MYOD1	2392	0.009
ENSP00000279387	PPP4C	482	0.009
ENSP00000296028	PPBP	3	0.009
ENSP00000320378	SLC7A8	474	0.009
ENSP00000342278	OAS2	481	0.009
ENSP00000367802	TAF1C	482	0.009
ENSP00000415183	MUC2	482	0.009
ENSP00000228837	FGF6	1434	0.01
ENSP00000230050	RPS12	481	0.01
ENSP00000236147	SELL	482	0.01
ENSP00000306866	GABARAP	482	0.01
ENSP00000276414	GNRH1	962	0.011
ENSP00000304669	CTNNA1	969	0.011
ENSP00000346879	NKX2-1	482	0.011
ENSP00000361965	ADA	482	0.011
ENSP00000364737	CENPP	481	0.011
ENSP00000387662	GCG	1444	0.011
ENSP00000304408	COL3A1	482	0.012

ENSP00000304915	IL13	482	0.012
ENSP00000294304	LRP5	482	0.013
ENSP00000312017	FAM57A	1	0.013
ENSP00000242067	BBS9	482	0.014
ENSP00000338345	SNCA	1217	0.014
ENSP00000244926	SCGB1D2	481	0.015
ENSP00000265773	SMARCA2	962	0.015
ENSP00000268057	BBS4	474	0.016
ENSP00000315130	CLU	481	0.016
ENSP00000356024	CR2	667	0.016
ENSP00000360025	GADD45A	482	0.017
ENSP00000355759	PARP1	1441	0.018
ENSP00000222382	CYP3A43	89	0.019
ENSP00000256857	GRP	975	0.019
ENSP00000346032	ANXA2	482	0.019
ENSP00000374280	RTF1	482	0.019
ENSP00000216267	BRD1	1	0.02
ENSP00000221265	PAF1	850	0.02
ENSP00000260682	CYP2C9	502	0.02
ENSP00000367316	ITGA8	1144	0.02
ENSP00000168712	FGF4	6	0.021
ENSP00000268171	FURIN	482	0.021

ENSP00000338799	IL6ST	1126	0.022
ENSP00000363548	CXCL12	482	0.022
ENSP00000225698	C1QBP	482	0.023
ENSP00000305422	CEBPB	220	0.023
ENSP00000308208	MMP14	4	0.023
ENSP00000365435	TNFRSF1B	482	0.023
ENSP00000367299	VPS36	482	0.023
ENSP00000296785	ANKRA2	13	0.024
ENSP00000363089	TLR4	962	0.024
ENSP00000309968	ADAM17	480	0.025
ENSP00000342143	PDZK1	481	0.025
ENSP00000349887	ASNA1	478	0.025
ENSP00000231509	NR3C1	2843	0.026
ENSP00000296674	RPS23	457	0.026
ENSP00000355518	FH	304	0.027
ENSP00000227752	IL10RA	482	0.029
ENSP00000407375	GPX1	482	0.03
ENSP00000259512	DERL1	482	0.031
ENSP00000300161	YWHAB	958	0.031
ENSP00000356070	MAPKAPK2	961	0.031
ENSP00000003100	CYP51A1	1	0.032
ENSP00000262965	TCF3	964	0.032

ENSP00000300134	STAT6	1291	0.032
ENSP00000155840	KCNQ1	482	0.033
ENSP00000263734	EPAS1	39	0.033
ENSP00000342952	ADCY2	1059	0.033
ENSP00000363998	ITCH	955	0.033
ENSP00000256078	KRAS	341	0.034
ENSP00000287820	PPARG	2238	0.034
ENSP00000296509	MAD2L1	482	0.034
ENSP00000419879	CDS2	1	0.035
ENSP00000338964	GGT7	1	0.036
ENSP00000373614	SELPLG	482	0.036
ENSP00000365682	TLE1	482	0.037
ENSP00000229595	ASF1A	482	0.038
ENSP00000240618	KLRK1	482	0.039
ENSP00000261167	WBP11	1	0.039
ENSP00000294728	VCAM1	14	0.039
ENSP00000308845	MRPS12	1	0.039
ENSP00000332353	PTCH1	1438	0.039
ENSP00000220809	PLAT	482	0.04
ENSP00000245907	C3	962	0.04
ENSP00000331736	SELE	1	0.04
ENSP00000283131	SMARCA5	482	0.041

ENSP00000312652	LEP	1360	0.041
ENSP00000324648	CYP2B6	509	0.043
ENSP00000359998	GSTA4	10	0.043
ENSP00000316054	DVL3	482	0.044
ENSP00000329967	TBK1	951	0.044
ENSP00000352272	MYOZ1	481	0.044
ENSP00000262077	NUP153	482	0.045
ENSP00000263645	CD81	36	0.045
ENSP00000297268	COL1A2	482	0.045
ENSP00000268296	ITGAX	6	0.046
ENSP00000351407	ARNT	1300	0.046
ENSP00000010338	TRAF3IP3	482	0.047
ENSP00000248244	TICAM1	480	0.047
ENSP00000331487	NPLOC4	473	0.047
ENSP00000320180	GHRHR	472	0.048
ENSP00000338018	HIF1A	6169	0.048
ENSP00000340944	PTPN11	1219	0.048
ENSP00000366565	VPS28	482	0.049
ENSP00000290649	AMFR	482	0.05
ENSP00000251772	PLXNA1	1	0.051
ENSP00000300589	NOD2	3	0.051
ENSP00000170630	IL4R	815	0.052

ENSP00000232458	ECT2	5	0.052
ENSP00000236850	APOA1	1846	0.053
ENSP00000216181	MYH9	1	0.054
ENSP00000264708	POMC	1847	0.054
ENSP00000295731	IHH	8	0.054
ENSP00000310551	LCLAT1	1	0.056
ENSP00000354612	PTGS1	648	0.056
ENSP00000303242	ITGB2	1356	0.057
ENSP00000316176	UBE2N	482	0.057
ENSP00000250416	PARP2	481	0.058
ENSP00000311364	MUCL1	481	0.058
ENSP00000326550	TACC3	482	0.058
ENSP00000226218	VTN|SEBOX	50	0.06
ENSP00000382342	ABCC1	461	0.062
ENSP00000220592	AGO2	408	0.063
ENSP00000227918	SCGB2A2	481	0.063
ENSP00000263686	SELP	957	0.064
ENSP00000282441	YAP1	482	0.064
ENSP00000301149	GPD1	2	0.064
ENSP00000262133	RBL2	6	0.065
ENSP00000273430	AGTR1	929	0.065
ENSP00000287497	ITGAM	888	0.066

ENSP00000295683	CXCR1	3	0.067
ENSP00000221930	TGFB1	1758	0.068
ENSP00000339007	GRB2	5685	0.068
ENSP00000216797	NFKBIA	552	0.069
ENSP00000355231	BECN1	482	0.069
ENSP00000260731	KIF11	482	0.071
ENSP00000380349	CAPN3	12	0.071
ENSP00000264968	MGAT4A	9	0.072
ENSP00000324856	STK11	482	0.073
ENSP00000333194	RGS19	3	0.074
ENSP00000360372	CYP2C19	122	0.074
ENSP00000300651	MED1	992	0.075
ENSP00000245919	FOSB	277	0.076
ENSP00000362166	MEAF6	481	0.078
ENSP00000254480	SMARCC1	736	0.079
ENSP00000253401	ARHGEF9	1	0.081
ENSP00000261973	RBM25	480	0.081
ENSP00000264499	BBS7	1	0.083
ENSP00000250617	ARHGEF6	339	0.085
ENSP00000253188	SLC7A10	6	0.086
ENSP00000293379	ITGA5	1893	0.086
ENSP00000348784	IGBP1	482	0.086

ENSP00000335153	HSP90AA1	17385	0.087
ENSP00000384273	RELA	1745	0.087
ENSP00000250448	FOXA1	479	0.091
ENSP00000253024	TRIM28	482	0.091
ENSP00000302021	MUC7	1	0.092
ENSP00000314949	POLR2A	1939	0.092
ENSP00000285930	AKR1B1	430	0.093
ENSP00000260356	THBS1	10	0.094
ENSP00000281453	CENPU	481	0.094
ENSP00000211122	GSTA3	2	0.096
ENSP00000364094	ITGB1	2172	0.097
ENSP00000369213	DDX58	1187	0.099
ENSP00000341268	TRADD	9	0.101
ENSP00000358301	ADRB1	12	0.101
ENSP00000222254	PIK3R2	6	0.103
ENSP00000340698	GIPC1	444	0.103
ENSP00000342056	CS	1	0.103
ENSP00000343204	JAK1	1161	0.103
ENSP00000359206	BTRC	207	0.103
ENSP00000294339	TAL1	482	0.104
ENSP00000392423	RELN	369	0.104
ENSP00000216223	IL2RB	646	0.105

ENSP00000306397	UQCRFS1	30	0.105
ENSP00000221130	GSR	3	0.109
ENSP00000278823	MTA2	1	0.109
ENSP00000400591	SNRPE	122	0.109
ENSP00000390500	STK3	362	0.11
ENSP00000264554	SHC2	424	0.111
ENSP00000361125	VEGFA	1442	0.111
ENSP00000358674	UBL4A	478	0.113
ENSP00000362413	PGK1	472	0.113
ENSP00000384515	PARVB	339	0.117
ENSP00000253363	RBM39	480	0.119
ENSP00000285379	CA2	482	0.119
ENSP00000303634	LRP8	369	0.119
ENSP00000313419	CD19	701	0.119
ENSP00000364133	TGFBR1	1707	0.119
ENSP00000262445	MAP2K4	201	0.122
ENSP00000351997	MAP2K6	419	0.122
ENSP00000379213	PTHLH	8	0.122
ENSP00000361423	ABL1	836	0.123
ENSP00000397552	ACTL6A	486	0.124
ENSP00000265038	ERCC8	12	0.129
ENSP00000225983	HDAC5	8	0.13

ENSP00000360217	RHAG	130	0.13
ENSP00000371973	SAP18	480	0.13
ENSP00000259075	TANK	8	0.132
ENSP00000286301	CSF1R	476	0.135
ENSP00000371067	JAK2	1809	0.137
ENSP00000259808	RIPK1	817	0.138
ENSP00000382004	CTNND1	681	0.138
ENSP00000314897	ANGPT2	1	0.139
ENSP00000274459	ATG12	480	0.14
ENSP00000290158	KPNB1	482	0.142
ENSP00000374372	SPTB	352	0.142
ENSP00000312999	GNAI2	3	0.143
ENSP00000225792	DDX5	1	0.144
ENSP00000243776	CHPF	479	0.144
ENSP00000329411	IRF7	18	0.145
ENSP00000315011	EDNRA	1	0.146
ENSP00000383977	TEK	1	0.146
ENSP00000327070	MDH2	42	0.147
ENSP00000245414	IRF1	481	0.148
ENSP00000346437	ATG7	480	0.149
ENSP00000225577	RPS6KB1	482	0.151
ENSP00000312697	DMAP1	481	0.152

ENSP00000358716	DDX20	408	0.152
ENSP00000379339	RPS29	24	0.152
ENSP00000346389	MEF2A	10	0.153
ENSP00000356425	UCHL5	482	0.153
ENSP00000358525	NGF	1291	0.153
ENSP00000252945	CYP2E1	481	0.154
ENSP00000261799	PDGFRB	676	0.155
ENSP00000360683	PTPN1	598	0.157
ENSP00000283006	CENPH	1	0.158
ENSP00000358997	IRAK1	1426	0.16
ENSP00000350720	SMARCA4	1223	0.161
ENSP00000360916	VAV2	424	0.162
ENSP00000367309	MAOB	6	0.162
ENSP00000366607	SF1	8	0.163
ENSP00000220751	RIPK2	3	0.167
ENSP00000263621	ELANE	3	0.167
ENSP00000308533	GEMIN2	408	0.169
ENSP00000360869	IFIT1	471	0.175
ENSP00000301838	FADD	375	0.176
ENSP00000401980	MAVS	483	0.178
ENSP00000355632	GALNT2	2	0.18
ENSP00000358335	MAP3K7	419	0.182

ENSP00000274376	RASA1	727	0.183
ENSP00000302269	VAV1	670	0.184
ENSP00000267082	ITGB7	243	0.186
ENSP00000354859	DRD2	132	0.186
ENSP00000273951	GC	1	0.19
ENSP00000372023	CHEK2	6	0.19
ENSP00000219476	TSC2	716	0.191
ENSP00000268035	IGF1R	1842	0.196
ENSP00000307272	RPTOR	2	0.196
ENSP00000263464	BIRC3	3	0.198
ENSP00000337103	CHAT	8	0.199
ENSP00000360519	RBP4	460	0.199
ENSP00000254066	RARA	1332	0.2
ENSP00000342007	CYP1A2	198	0.2
ENSP00000229794	MAPK14	956	0.201
ENSP00000262435	SMURF2	12	0.202
ENSP00000252486	APOE	369	0.203
ENSP00000330393	LEPR	7	0.208
ENSP00000387286	RAB1A	482	0.209
ENSP00000222005	CDC37	538	0.21
ENSP00000413254	RPH3A	1	0.211
ENSP00000297185	HSPA9	482	0.213

ENSP00000358541	SIKE1	105	0.214
ENSP00000363512	ALOX5	8	0.215
ENSP00000358866	FLNA	430	0.216
ENSP00000233146	MSH2	481	0.22
ENSP00000223095	SERPINE1	567	0.227
ENSP00000360718	RAB3B	1	0.232
ENSP00000318585	BACE1	480	0.233
ENSP00000265023	KNG1	480	0.235
ENSP00000261707	SLC6A4	34	0.242
ENSP00000280097	HNMT	154	0.242
ENSP00000360266	JUN	4118	0.243
ENSP00000368699	ISG15	708	0.243
ENSP00000238081	YWHAQ	1	0.247
ENSP00000287936	HMGCR	2644	0.247
ENSP00000261023	ITGAV	101	0.249
ENSP00000363641	TXN	18	0.25
ENSP00000414303	BDNF	54	0.251
ENSP00000374354	EXOSC8	575	0.252
ENSP00000348551	NCOR2	502	0.256
ENSP00000380227	ITGA4	411	0.256
ENSP00000354522	TOP1	242	0.26
ENSP00000304895	IRS1	1402	0.263

ENSP00000356087	IKBKE	94	0.263
ENSP00000264033	CBL	9217	0.266
ENSP00000228682	GLI1	4	0.267
ENSP00000310127	IRF3	264	0.268
ENSP00000196551	RPS5	1	0.27
ENSP00000265724	ABCB1	35	0.272
ENSP00000368683	EDN1	93	0.272
ENSP00000260433	CYP19A1	483	0.273
ENSP00000338934	EZR	1351	0.273
ENSP00000332643	NDN	53	0.276
ENSP00000222256	RAB3A	482	0.277
ENSP00000335620	GSTA1	87	0.278
ENSP00000370938	CDK8	357	0.278
ENSP00000044462	PSMA4	160	0.279
ENSP00000162749	TNFRSF1A	516	0.279
ENSP00000172229	NGFR	541	0.279
ENSP00000216911	AURKA	481	0.279
ENSP00000361892	STK4	3	0.28
ENSP00000233946	IL1R1	478	0.284
ENSP00000334122	FGF3	478	0.284
ENSP00000352257	XRCC6	1	0.285
ENSP00000354586	GLI2	2	0.289

ENSP00000419692	RXRA	554	0.294
ENSP00000252444	LDLR	8	0.296
ENSP00000321656	CDC25C	49	0.296
ENSP00000336630	ADORA2A	3	0.298
ENSP00000264110	ATF2	387	0.3
ENSP00000209875	CBX5	482	0.305
ENSP00000303019	GPHN	2	0.308
ENSP00000324729	SAV1	3	0.312
ENSP00000211998	VCL	474	0.313
ENSP00000300413	SNRPD1	408	0.313
ENSP00000358595	CGA	478	0.313
ENSP00000247668	TRAF2	1422	0.314
ENSP00000265351	XPO5	74	0.314
ENSP00000239223	DUSP1	124	0.317
ENSP00000262613	SLC9A3R1	1340	0.318
ENSP00000265709	ANK1	352	0.318
ENSP00000225964	COL1A1	482	0.319
ENSP00000278616	ATM	1219	0.319
ENSP00000256104	FABP4	1	0.327
ENSP00000326804	CUL1	482	0.331
ENSP00000349437	IGF2R	142	0.331
ENSP00000372793	LTA	5	0.331

ENSP00000229854	MCM3	351	0.333
ENSP00000354720	SMC3	479	0.337
ENSP00000358918	SUFU	3	0.342
ENSP00000318297	RUVBL1	478	0.348
ENSP00000350275	HIST1H3A	482	0.349
ENSP00000251849	RAF1	5554	0.355
ENSP00000317159	CYC1	30	0.356
ENSP00000355261	SMG5	9	0.367
ENSP00000254940	NIP7	482	0.368
ENSP00000261205	SYT1	481	0.368
ENSP00000341551	SMAD4	1007	0.368
ENSP00000386896	ITGA6	5	0.369
ENSP00000228307	PXN	419	0.371
ENSP00000282561	GJA1	480	0.373
ENSP00000362361	CDK9	593	0.374
ENSP00000269260	ARRB2	957	0.375
ENSP00000311113	JUP	289	0.38
ENSP00000287727	ZFYVE9	464	0.382
ENSP00000348307	SIRPA	264	0.383
ENSP00000311677	PPP1R8	481	0.384
ENSP00000353483	MAPK8	783	0.386
ENSP00000347232	BLM	1	0.387

ENSP00000268712	NCOR1	397	0.388
ENSP00000212015	SIRT1	46	0.391
ENSP00000271452	NUF2	1	0.4
ENSP00000351908	MAP3K5	18	0.405
ENSP00000384708	FSHR	2	0.408
ENSP00000276198	HTR2C	5	0.414
ENSP00000340820	MAPT	482	0.418
ENSP00000359074	L1CAM	352	0.421
ENSP00000254122	FSHB	2	0.424
ENSP00000223321	PSMA2	321	0.427
ENSP00000215071	PSMD8	6	0.435
ENSP00000301141	CYP2A6	1	0.435
ENSP00000396219	MEF2C	5	0.443
ENSP00000341344	GGA1	480	0.449
ENSP00000355325	PSMB5	160	0.449
ENSP00000361850	PLAU	128	0.45
ENSP00000356438	PTGS2	687	0.453
ENSP00000311344	PPP2R1B	27	0.455
ENSP00000249299	NAA38	61	0.46
ENSP00000352980	HIST1H4A	487	0.465
ENSP00000354394	STAT1	841	0.468
ENSP00000333982	NDEL1	1	0.472

ENSP00000271628	SF3B4	9	0.475
ENSP00000296581	LSM6	3	0.478
ENSP00000340691	EIF4EBP1	324	0.481
ENSP00000296585	ITGA2	529	0.487
ENSP00000315644	TYMS	489	0.489
ENSP00000264606	HDAC4	5	0.496
ENSP00000342374	SNRPD2	41	0.499
ENSP00000351273	CASP8	118	0.51
ENSP00000297338	RAD21	7	0.511
ENSP00000256443	CDK7	14	0.512
ENSP00000352608	RYR1	16	0.513
ENSP00000299421	ILK	347	0.514
ENSP00000292303	CCR5	316	0.516
ENSP00000371138	FKBP1A	20	0.52
ENSP00000308938	PLG	713	0.523
ENSP00000272190	REN	39	0.525
ENSP00000372975	HLA-C	5	0.529
ENSP00000315859	RNPS1	138	0.531
ENSP00000349467	CALM1	482	0.537
ENSP00000306245	FOS	1654	0.539
ENSP00000354558	MTOR	1071	0.541
ENSP00000332468	TRAF3	471	0.543

ENSP00000209728	CDC6	385	0.549
ENSP00000319635	CXCR2	3	0.551
ENSP00000267868	RAD51	247	0.562
ENSP00000359531	GTF2B	137	0.564
ENSP00000337825	LCK	1307	0.567
ENSP00000367797	SKI	3	0.567
ENSP00000379625	MYD88	478	0.567
ENSP00000269305	TP53	15682	0.568
ENSP00000278572	RPS3	1	0.571
ENSP00000358622	IKBKG	3	0.575
ENSP00000359345	RPL5	973	0.577
ENSP00000341189	PTK2	1217	0.578
ENSP00000384675	SOS1	1378	0.579
ENSP00000301280	CHAF1A	487	0.581
ENSP00000305372	ADRB2	808	0.581
ENSP00000299543	CTDP1	184	0.587
ENSP00000386259	NEB	12	0.592
ENSP00000250495	NEDD8	85	0.593
ENSP00000363071	DES	12	0.598
ENSP00000343274	INTS8	94	0.6
ENSP00000396308	DHFR	3	0.6
ENSP00000261405	VWF	49	0.601

ENSP00000370571	TH	2	0.601
ENSP00000353622	SIN3A	339	0.604
ENSP00000344818	UBC	42741	0.609
ENSP00000370473	IGFBP3	678	0.609
ENSP00000221138	PPP2CB	12	0.611
ENSP00000409132	HLA-G	211	0.611
ENSP00000262629	TYROBP	742	0.616
ENSP00000301019	CDT1	15	0.618
ENSP00000309103	BAD	105	0.623
ENSP00000314004	ANAPC2	12	0.633
ENSP00000295897	ALB	211	0.64
ENSP00000256442	CCNB1	4879	0.646
ENSP00000315702	MOB4	148	0.646
ENSP00000262160	SMAD2	577	0.647
ENSP00000278916	CHEK1	37	0.647
ENSP00000268058	PML	493	0.648
ENSP00000257181	PRPF38A	3	0.656
ENSP00000291552	U2AF1	112	0.656
ENSP00000274335	PIK3R1	1253	0.657
ENSP00000302961	HSPA4	16	0.665
ENSP00000262477	RABEP1	338	0.668
ENSP00000366135	EXOSC10	2032	0.669

ENSP00000046794	LCP2	131	0.672
ENSP00000242577	DYNLL1	310	0.672
ENSP00000297494	NOS3	622	0.673
ENSP00000302967	HDAC3	55	0.674
ENSP00000264381	BCHE	1437	0.677
ENSP00000267101	ERBB3	9	0.677
ENSP00000215829	SNRPD3	626	0.684
ENSP00000324804	PPP2R1A	32	0.696
ENSP00000284957	RABGEF1	338	0.697
ENSP00000230354	TBP	612	0.701
ENSP00000308450	CDC20	655	0.705
ENSP00000396127	RAN	949	0.707
ENSP00000364898	SYK	763	0.71
ENSP00000266000	DAXX	2	0.712
ENSP00000260970	PPIG	3	0.713
ENSP00000348577	RANGAP1	929	0.72
ENSP00000219548	STUB1	405	0.724
ENSP00000287647	FANCD2	17	0.728
ENSP00000297151	SLU7	3	0.731
ENSP00000240185	TARDBP	1967	0.745
ENSP00000418447	PPP2CA	875	0.747
ENSP00000353059	APAF1	181	0.751

ENSP00000215832	MAPK1	758	0.754
ENSP00000297518	CDK5	1	0.757
ENSP00000234111	ODC1	4	0.759
ENSP00000381331	HDAC2	115	0.76
ENSP00000261461	PPP2R5A	93	0.763
ENSP00000263753	SGOL1	768	0.768
ENSP00000339109	ANAPC1	371	0.768
ENSP00000308541	F2	396	0.772
ENSP00000302564	BCL2L1	26	0.774
ENSP00000329380	GP1BA	271	0.774
ENSP00000302486	MAP2K1	521	0.775
ENSP00000262643	CCNE1	307	0.778
ENSP00000361418	IPO13	2	0.788
ENSP00000295797	PRKCI	4	0.792
ENSP00000278568	PAK1	101	0.793
ENSP00000216225	RBX1	272	0.796
ENSP00000363055	ZWINT	4	0.796
ENSP00000367959	HTR2A	1	0.797
ENSP00000288986	NCK1	101	0.798
ENSP00000362900	SRSF4	1	0.798
ENSP00000223023	WASL	459	0.799
ENSP00000262320	AXIN1	1065	0.799

ENSP00000302955	RRM2	24	0.8
ENSP00000311032	CASP3	236	0.802
ENSP00000309503	YWHAZ	336	0.807
ENSP00000369757	RPS6	10	0.809
ENSP00000326031	PPP1CA	1	0.813
ENSP00000235090	WDR77	736	0.823
ENSP00000258962	SRSF1	27	0.824
ENSP00000284811	TCEB1	10	0.826
ENSP00000301633	BIRC5	15	0.827
ENSP00000348986	INS-IGF2	601	0.829
ENSP00000340330	KAT5	484	0.835
ENSP00000222812	STX1A	459	0.837
ENSP00000378165	ZNF207	723	0.842
ENSP00000329357	SP1	159	0.843
ENSP00000359727	BAG2	3	0.845
ENSP00000301764	DDB1	12	0.846
ENSP00000362592	RBBP4	119	0.846
ENSP00000366013	GNB2L1	10	0.847
ENSP00000374455	SQSTM1	4	0.849
ENSP00000312735	POLR2B	550	0.85
ENSP00000354554	MT-CYB	30	0.851
ENSP00000310596	LSM1	32	0.852

ENSP00000378529	FZR1	26	0.852
ENSP00000300574	CRK	128	0.857
ENSP00000262238	YY1	1	0.86
ENSP00000357858	BUB3	723	0.866
ENSP00000299402	APBB1	1	0.867
ENSP00000361626	YBX1	18	0.87
ENSP00000312995	CLSPN	7	0.874
ENSP00000011653	CD4	321	0.879
ENSP00000284981	APP	3	0.88
ENSP00000368104	BMP2	46	0.887
ENSP00000348554	CDC16	14	0.888
ENSP00000231487	SKP1	213	0.89
ENSP00000227758	BIRC2	22	0.895
ENSP00000262803	UPF1	28	0.898
ENSP00000346022	RPL9	1	0.903
ENSP00000324806	GSK3B	329	0.915
ENSP00000260363	KIF23	1	0.917
ENSP00000269141	CDH2	2201	0.918
ENSP00000376076	SUMO1	70	0.922
ENSP00000324897	UBE2I	859	0.93
ENSP00000352400	NUP214	8	0.93
ENSP00000314214	VAMP2	22	0.935

ENSP00000362820	SRSF3	2	0.939
ENSP00000262187	RHEB	1	0.94
ENSP00000354961	MT-ND4	16	0.94
ENSP00000294172	NXF1	8	0.941
ENSP00000263309	CLNS1A	274	0.947
ENSP00000292644	PSMC2	2012	0.947
ENSP00000319169	PRMT5	275	0.949
ENSP00000414634	LSM2	64	0.954
ENSP00000219255	PARD6A	4	0.955
ENSP00000354876	MT-CO2	14	0.955
ENSP00000313950	AURKB	11	0.957
ENSP00000354499	MT-CO1	14	0.957
ENSP00000280665	DCP1B	1	0.959
ENSP00000352516	DNMT1	440	0.961
ENSP00000350877	SRSF2	2	0.962
ENSP00000221494	SF3A2	919	0.964
ENSP00000269349	EIF4A3	22	0.967
ENSP00000264951	XRN1	12	0.969
ENSP00000252102	NDUFA2	16	0.97
ENSP00000274026	CCNA2	1444	0.97
ENSP00000248566	SHFM1	675	0.971
ENSP00000307863	U2AF2	978	0.971

ENSP00000314458	CDC42	1120	0.972
ENSP00000003084	CFTR	11652	0.973
ENSP00000281708	FBXW7	24	0.975
ENSP00000162330	BCAR1	169	0.977
ENSP00000353224	TFRC	89	0.977
ENSP00000417404	HFE	89	0.977
ENSP00000340858	B2M	211	0.979
ENSP00000363676	RPL11	451	0.979
ENSP00000316879	EIF4G1	22	0.981
ENSP00000348708	UPF2	59	0.981
ENSP00000276201	UPF3B	36	0.982
ENSP00000365891	WAS	86	0.984
ENSP00000326366	PSEN1	30	0.987
ENSP00000367207	MYC	16	0.988
ENSP00000357656	FYN	324	0.989
ENSP00000314491	SRRT	77	0.99
ENSP00000268182	IQGAP1	17	0.991
ENSP00000287598	BUB1B	395	0.992
ENSP00000365439	HNRNPK	970	0.993
ENSP00000398597	EXOSC6	6	0.993
ENSP00000227378	HSPA8	80	0.996
ENSP00000313829	KHDRBS1	953	0.996

ENSP00000348461	RAC1	14	0.998
CID000004740	pentoxifylline	6	0
CID000017734	HC Blue 1	1	0
CID000024841	iodide	2642	0
CID000107921	S-mephenytoin	482	0
CID000161491	MMA(III	482	0
CID000440994	lacto-N-biose	482	0
CID000441248	Gal beta 1-3GalNAc	482	0
CID000007628	borate	482	0.001
CID000008299	acetol	480	0.001
CID000065064	EGCG	482	0.001
CID002733525	tamoxifen	4379	0.001
CID000000890	phytate	482	0.002
CID000004594	omeprazole	482	0.002
CID000097535	NSC638615	1	0.002
CID000000880	methylglyoxal	479	0.004
CID000008582	inosine monophosphate	482	0.004
CID000024139	chitin	2839	0.004
CID000000180	acetone	1	0.006
CID000000525	malate	479	0.006
CID006433205	methylazoxymethanol	480	0.006
CID000006106	L-leucine	955	0.007

CID000003559	haloperidol	482	0.008
CID000006359	BDCM	481	0.008
CID000010461	sulfur mustard	482	0.008
CID000124886	Glutathione	1433	0.01
CID006326970	selenium	482	0.01
CID000007845	1,3-butadiene	1	0.011
CID000000949	N,N-dimethylaniline	478	0.013
CID000006503	tris	1440	0.013
CID000051683	swainsonine	481	0.013
CID000392622	ritonavir	480	0.02
CID000000978	p-aminobenzoic a	481	0.022
CID000060749	gemcitabine	348	0.022
CID000092824	D-malate	1	0.022
CID000002733	chlorzoxazone	2	0.024
CID000000312	Chloride ion	4870	0.026
CID000440552	beta-GalNAc	478	0.026
CID000002771	citalopram	26	0.028
CID000006115	aniline	1	0.03
CID000001032	propionate	478	0.031
CID000006167	colchicine	482	0.031
CID000043815	paroxetine	5	0.033
CID000008947	monomethylarsonic acid	2	0.034

CID000010214	3'-phosphoadenosine 5'-phosphosulfate	482	0.036
CID000439162	alpha-glycerophosphate	763	0.036
CID000003032	diclofenac	489	0.037
CID000000768	hydrogen cyanide	481	0.038
CID000172197	angiotensin II	448	0.039
CID000000170	dihydropteroate	1	0.045
CID000002514	cadmium	482	0.05
CID000000781	thiocyanate	478	0.053
CID000007405	pyroglutamate	480	0.053
CID000000936	vitamin B	39	0.054
CID000002513	dimethylarsinic a	1	0.058
CID000002541	candesartan	7	0.059
CID000003776	isopropanol	3	0.059
CID023724461	UDP-N-acetyl-alpha-D-galactosamine	482	0.06
CID000065999	telmisartan	916	0.066
CID000439427	Glc-6-P	1	0.069
CID000004174	metyrapone	11	0.07
CID000005754	cortisol	1598	0.07
CID000033032	glutamic a	1932	0.07
CID000060699	topotecan	242	0.07
CID000003961	losartan	39	0.071
CID000444972	fumarate	262	0.072

CID000061503	D-lactate	46	0.073
CID000002244	aspirin	68	0.077
CID000643975	flavin adenine dinucleotide	1108	0.077
CID000005743	dexamethasone	724	0.085
CID000001775	phenytoin	8	0.088
CID011020241	ascorbate	2	0.094
CID000005472	ticlopidine	31	0.104
CID000000970	oxaloacetate	480	0.105
CID000004829	pioglitazone	104	0.105
CID000055245	mifepristone	479	0.107
CID000071077	D-serine	6	0.116
CID000000177	acetaldehyde	947	0.124
CID000000784	H2O2	1436	0.124
CID000000760	glyoxylate	234	0.137
CID000005997	cholesterol	2654	0.14
CID000008629	UDP-glucose	2	0.146
CID000003016	diazepam	24	0.154
CID000445354	retinol	461	0.154
CID005280453	1,25(OH)2D3	482	0.155
CID000643976	flavin mononucleotide	248	0.157
CID000013945	dCMP	4	0.167
CID000002336	benzo(a)pyrene	1	0.176

CID000000647	DMAPP	154	0.19
CID000000887	methanol	34	0.19
CID000005819	thyroxine	56	0.193
CID000001003	phosphate	2042	0.201
CID000006140	phenylalanine	38	0.202
CID000005815	epinephrine	1	0.221
CID000001935	tacrine	172	0.223
CID005283731	25-hydroxyvitamin D	1	0.228
CID000071080	D-alanine	6	0.231
CID000031100	bromocriptine	2	0.243
CID000002519	caffeine	21	0.244
CID000145742	L-proline	4	0.246
CID000638015	11-cis-retinal	21	0.247
CID000005961	glutamine	461	0.264
CID000000712	formalin	4	0.272
CID000002520	verapamil	33	0.287
CID000002332	benzamidine	1	0.297
CID000006137	L-methionine	3	0.315
CID000000668	dihydroxyacetone phosphate	4	0.321
CID000006131	poly C	482	0.324
CID000006022	adenosine diphosphate	2121	0.329
CID000098792	dihydrofolate	1	0.339

CID000004946	propranolol	393	0.355
CID000005994	progesterone	3	0.356
CID000446220	cocaine	11	0.361
CID000005757	estradiol	496	0.368
CID000439260	norepinephrine	508	0.372
CID000065063	dUMP	12	0.381
CID000001567	2-mercaptoethanol	4	0.398
CID000006031	uridine diphosphate	954	0.399
CID000016230	amiloride	38	0.4
CID000006128	androstenedione	1	0.423
CID000000991	parathion	1444	0.43
CID000002818	clozapine	34	0.433
CID000060961	adenosine	484	0.44
CID000005950	L-alanine	20	0.443
CID000005881	DHEA	1	0.451
CID000000813	potassium	482	0.503
CID000006322	arginine	638	0.527
CID000000888	magnesium	4251	0.551
CID005280360	PGE2	115	0.552
CID000000679	DMSO	47	0.554
CID000000280	CO(2	282	0.564
CID000000767	bicarbonate	197	0.574

CID000006076	cyclic AMP	482	0.578
CID000006030	uridylate	414	0.592
CID000000774	histamine	701	0.595
CID000000681	dopamine	383	0.609
CID000006013	testosterone	656	0.616
CID000001102	spermidine	3	0.62
CID000089594	nicotine	3	0.624
CID000000977	oxygen	3591	0.633
CID000000222	ammonia	130	0.652
CID000006804	5'-GMP	62	0.659
CID000444899	arachidonic a	353	0.666
CID000000750	glycine	522	0.671
CID000001195	isopentenyl diphosphate	154	0.686
CID000003779	isoproterenol	4	0.705
CID000445675	UDP-GlcNAc	9	0.706
CID000000051	alpha-ketoglutarate	523	0.712
CID000000187	acetylcholine	259	0.746
CID000004585	olanzapine	10	0.746
CID000006029	uridine	8	0.763
CID000005202	serotonin	39	0.775
CID000066370	alpha-D-glucose	3	0.792
CID000001051	pyridoxal 5'-phosphate	475	0.798

CID000000305	choline	1385	0.807
CID000006083	adenosine monophosphate	490	0.821
CID000000923	sodium	2464	0.826
CID000005962	lysine	11	0.839
CID000000985	palmitate	1	0.929
CID000000283	formate	280	0.934
CID000000961	hydroxyl radicals	613	0.949
CID000439155	AdoHcy	445	0.954
CID000000783	hydride	4	0.955
CID000005885	NADP	529	0.96
CID000005957	adenosine triphosphate	27023	0.966
CID000023925	Fe(III	767	0.966
CID000000190	adenine	7	0.978
CID000000753	glycerol	2505	0.979
CID000023930	manganese	15	0.983
CID000005892	beta-NAD	87	0.996
CID000087642	coenzyme A	8	0.996
CID000023994	zinc	3280	0.997
CID000000271	calcium	12484	0.998
CID000006830	guanosine triphosphate	4192	1
CID000008977	guanosine diphosphate	497	1
