# Supplementary material for: Identification of Novel Thyroid Cancer-Related Genes and Chemicals Using Shortest Path Algorithm
Source: Biomed Res Int. 2015 Mar 22;2015:964795. doi: 10.1155/2015/964795 (PMC4385622; doi:10.1155/2015/964795)
Supplement: Supplementary file 1 — The Supplementary Material contains six files. In detail, Online Supporting Information S1 lists thyroid cancer-related genes and chemicals; Online Supporting Information S2 lists 636 candidate genes and 174 candidate chemicals; Online Supporting Information S3 lists 169 significant candidate genes and 49 significant candidate chemicals; Online Supporting Information S4 lists KEGG enrichment results of 169 significant candidate genes; Online Supporting Information S5 lists GO enrichment results of 169 significant candidate genes; Online Supporting Information S6 lists the discussion of 29 significant candidate chemicals. [file 964795.f1.zip › Supp-S3.pdf]

Online Supporting Information S3. 169 significant candidate genes and 49 significant candidate chemicals with permutation FDRs smaller than 0.05.

**Gene or chemical ID**	**Gene or chemical name**	**Betweenness**	**Permutation FDR**
ENSP00000206249	ESR1	21841	0
ENSP00000226574	NFKB1	963	0
ENSP00000227507	CCND1	6321	0
ENSP00000259526	NOV	482	0
ENSP00000263851	IL7	482	0
ENSP00000268655	ZNF174	1	0
ENSP00000269138	SS18	963	0
ENSP00000295108	NEUROD1	482	0
ENSP00000301012	MVD	482	0
ENSP00000302640	UBTF	482	0
ENSP00000302648	NRTN	482	0
ENSP00000304811	UGT2B7	963	0
ENSP00000306157	IL7R	482	0
ENSP00000327213	NRIP1	963	0
ENSP00000334181	KDM4D	482	0
ENSP00000337915	CYP3A4	4122	0
ENSP00000349508	CHD4	482	0
ENSP00000360973	AGTR2	482	0
ENSP00000368066	HAO1	482	0

ENSP00000368981	SLC35B3	482	0
ENSP00000377941	ACTN1	962	0
ENSP00000262809	ELL	482	0.001
ENSP00000270349	SLC6A3	956	0.001
ENSP00000295408	MERTK	962	0.001
ENSP00000338207	LMO1	482	0.001
ENSP00000346015	RPL27A	477	0.001
ENSP00000348089	ERCC6	482	0.001
ENSP00000357255	BGLAP	482	0.001
ENSP00000368754	BAZ2A	482	0.001
ENSP00000369396	SLC4A11	482	0.001
ENSP00000416387	FBLIM1	43	0.001
ENSP00000011684	PLEKHG6	482	0.002
ENSP00000262367	CREBBP	5560	0.002
ENSP00000276297	DLC1	482	0.002
ENSP00000327246	VIPR1	476	0.002
ENSP00000331358	GAST	997	0.002
ENSP00000335544	CCKBR	696	0.002
ENSP00000351605	FZD6	482	0.002
ENSP00000362690	NR5A1	482	0.002
ENSP00000371532	VLDLR	494	0.002
ENSP00000380514	HAGH	3	0.002

ENSP00000391998	ARTN	482	0.002
ENSP00000397297	NTF3	484	0.002
ENSP00000274565	SPINK7	482	0.003
ENSP00000290330	SNF8	482	0.003
ENSP00000370748	IDI1	482	0.003
ENSP00000417763	NAA10	482	0.003
ENSP00000253339	LATS1	482	0.004
ENSP00000331288	TMEM173	482	0.004
ENSP00000269485	TNFRSF11A	962	0.005
ENSP00000284818	LY96	962	0.005
ENSP00000323328	SIGLEC7	478	0.005
ENSP00000339151	IKBKB	501	0.005
ENSP00000344741	INSIG1	482	0.005
ENSP00000244007	PLCG1	2851	0.006
ENSP00000263208	HIRA	482	0.006
ENSP00000304169	PITX2	482	0.006
ENSP00000380033	DDX17	482	0.006
ENSP00000410294	FGFR2	964	0.006
ENSP00000227163	SPI1	482	0.007
ENSP00000279247	CAPN1	470	0.007
ENSP00000322142	ING5	482	0.007
ENSP00000401303	SHC1	2222	0.007

ENSP00000241052	CAT	483	0.008
ENSP00000245157	BBS2	954	0.008
ENSP00000346566	CKAP5	482	0.008
ENSP00000249373	SMO	482	0.009
ENSP00000250003	MYOD1	2392	0.009
ENSP00000279387	PPP4C	482	0.009
ENSP00000296028	PPBP	3	0.009
ENSP00000320378	SLC7A8	474	0.009
ENSP00000342278	OAS2	481	0.009
ENSP00000367802	TAF1C	482	0.009
ENSP00000415183	MUC2	482	0.009
ENSP00000228837	FGF6	1434	0.01
ENSP00000230050	RPS12	481	0.01
ENSP00000236147	SELL	482	0.01
ENSP00000306866	GABARAP	482	0.01
ENSP00000276414	GNRH1	962	0.011
ENSP00000304669	CTNNA1	969	0.011
ENSP00000346879	NKX2-1	482	0.011
ENSP00000361965	ADA	482	0.011
ENSP00000364737	CENPP	481	0.011
ENSP00000387662	GCG	1444	0.011
ENSP00000304408	COL3A1	482	0.012

ENSP00000304915	IL13	482	0.012
ENSP00000294304	LRP5	482	0.013
ENSP00000312017	FAM57A	1	0.013
ENSP00000242067	BBS9	482	0.014
ENSP00000338345	SNCA	1217	0.014
ENSP00000244926	SCGB1D2	481	0.015
ENSP00000265773	SMARCA2	962	0.015
ENSP00000268057	BBS4	474	0.016
ENSP00000315130	CLU	481	0.016
ENSP00000356024	CR2	667	0.016
ENSP00000360025	GADD45A	482	0.017
ENSP00000355759	PARP1	1441	0.018
ENSP00000222382	CYP3A43	89	0.019
ENSP00000256857	GRP	975	0.019
ENSP00000346032	ANXA2	482	0.019
ENSP00000374280	RTF1	482	0.019
ENSP00000216267	BRD1	1	0.02
ENSP00000221265	PAF1	850	0.02
ENSP00000260682	CYP2C9	502	0.02
ENSP00000367316	ITGA8	1144	0.02
ENSP00000168712	FGF4	6	0.021
ENSP00000268171	FURIN	482	0.021

ENSP00000338799	IL6ST	1126	0.022
ENSP00000363548	CXCL12	482	0.022
ENSP00000225698	C1QBP	482	0.023
ENSP00000305422	CEBPB	220	0.023
ENSP00000308208	MMP14	4	0.023
ENSP00000365435	TNFRSF1B	482	0.023
ENSP00000367299	VPS36	482	0.023
ENSP00000296785	ANKRA2	13	0.024
ENSP00000363089	TLR4	962	0.024
ENSP00000309968	ADAM17	480	0.025
ENSP00000342143	PDZK1	481	0.025
ENSP00000349887	ASNA1	478	0.025
ENSP00000231509	NR3C1	2843	0.026
ENSP00000296674	RPS23	457	0.026
ENSP00000355518	FH	304	0.027
ENSP00000227752	IL10RA	482	0.029
ENSP00000407375	GPX1	482	0.03
ENSP00000259512	DERL1	482	0.031
ENSP00000300161	YWHAB	958	0.031
ENSP00000356070	MAPKAPK2	961	0.031
ENSP00000003100	CYP51A1	1	0.032
ENSP00000262965	TCF3	964	0.032

ENSP00000300134	STAT6	1291	0.032
ENSP00000155840	KCNQ1	482	0.033
ENSP00000263734	EPAS1	39	0.033
ENSP00000342952	ADCY2	1059	0.033
ENSP00000363998	ITCH	955	0.033
ENSP00000256078	KRAS	341	0.034
ENSP00000287820	PPARG	2238	0.034
ENSP00000296509	MAD2L1	482	0.034
ENSP00000419879	CDS2	1	0.035
ENSP00000338964	GGT7	1	0.036
ENSP00000373614	SELPLG	482	0.036
ENSP00000365682	TLE1	482	0.037
ENSP00000229595	ASF1A	482	0.038
ENSP00000240618	KLRK1	482	0.039
ENSP00000261167	WBP11	1	0.039
ENSP00000294728	VCAM1	14	0.039
ENSP00000308845	MRPS12	1	0.039
ENSP00000332353	PTCH1	1438	0.039
ENSP00000220809	PLAT	482	0.04
ENSP00000245907	C3	962	0.04
ENSP00000331736	SELE	1	0.04
ENSP00000283131	SMARCA5	482	0.041

ENSP00000312652	LEP	1360	0.041
ENSP00000324648	CYP2B6	509	0.043
ENSP00000359998	GSTA4	10	0.043
ENSP00000316054	DVL3	482	0.044
ENSP00000329967	TBK1	951	0.044
ENSP00000352272	MYOZ1	481	0.044
ENSP00000262077	NUP153	482	0.045
ENSP00000263645	CD81	36	0.045
ENSP00000297268	COL1A2	482	0.045
ENSP00000268296	ITGAX	6	0.046
ENSP00000351407	ARNT	1300	0.046
ENSP00000010338	TRAF3IP3	482	0.047
ENSP00000248244	TICAM1	480	0.047
ENSP00000331487	NPLOC4	473	0.047
ENSP00000320180	GHRHR	472	0.048
ENSP00000338018	HIF1A	6169	0.048
ENSP00000340944	PTPN11	1219	0.048
ENSP00000366565	VPS28	482	0.049
CID000004740	pentoxifylline	6	0
CID000017734	HC Blue 1	1	0
CID000024841	iodide	2642	0
CID000107921	S-mephenytoin	482	0

CID000161491	MMA(III	482	0
CID000440994	lacto-N-biose	482	0
CID000441248	Gal beta 1-3GalNAc	482	0
CID000007628	borate	482	0.001
CID000008299	acetol	480	0.001
CID000065064	EGCG	482	0.001
CID002733525	tamoxifen	4379	0.001
CID000000890	phytate	482	0.002
CID000004594	omeprazole	482	0.002
CID000097535	NSC638615	1	0.002
CID000000880	methylglyoxal	479	0.004
CID000008582	inosine monophosphate	482	0.004
CID000024139	chitin	2839	0.004
CID000000180	acetone	1	0.006
CID000000525	malate	479	0.006
CID006433205	methylazoxymethanol	480	0.006
CID000006106	L-leucine	955	0.007
CID000003559	haloperidol	482	0.008
CID000006359	BDCM	481	0.008
CID000010461	sulfur mustard	482	0.008
CID000124886	Glutathione	1433	0.01
CID006326970	selenium	482	0.01

CID000007845	1,3-butadiene	1	0.011
CID000000949	N,N-dimethylaniline	478	0.013
CID000006503	tris	1440	0.013
CID000051683	swainsonine	481	0.013
CID000392622	ritonavir	480	0.02
CID000000978	p-aminobenzoic a	481	0.022
CID000060749	gemcitabine	348	0.022
CID000092824	D-malate	1	0.022
CID000002733	chlorzoxazone	2	0.024
CID000000312	Chloride ion	4870	0.026
CID000440552	beta-GalNAc	478	0.026
CID000002771	citalopram	26	0.028
CID000006115	aniline	1	0.03
CID000001032	propionate	478	0.031
CID000006167	colchicine	482	0.031
CID000043815	paroxetine	5	0.033
CID000008947	monomethylarsonic acid	2	0.034
CID000010214	3'-phosphoadenosine 5'-phosphosulfate	482	0.036
CID000439162	alpha-glycerophosphate	763	0.036
CID000003032	diclofenac	489	0.037
CID000000768	hydrogen cyanide	481	0.038
CID000172197	angiotensin II	448	0.039

CID000000170	dihydropteroate	1	0.045
