# Supplementary material for: Identification of Novel Thyroid Cancer-Related Genes and Chemicals Using Shortest Path Algorithm
Source: Biomed Res Int. 2015 Mar 22;2015:964795. doi: 10.1155/2015/964795 (PMC4385622; doi:10.1155/2015/964795)
Supplement: Supplementary file 1 — The Supplementary Material contains six files. In detail, Online Supporting Information S1 lists thyroid cancer-related genes and chemicals; Online Supporting Information S2 lists 636 candidate genes and 174 candidate chemicals; Online Supporting Information S3 lists 169 significant candidate genes and 49 significant candidate chemicals; Online Supporting Information S4 lists KEGG enrichment results of 169 significant candidate genes; Online Supporting Information S5 lists GO enrichment results of 169 significant candidate genes; Online Supporting Information S6 lists the discussion of 29 significant candidate chemicals. [file 964795.f1.zip › Supp-S6.pdf]

Online Supporting Information S6. Discussion of 29 significant candidate chemicals

1) 1,3-butadiene (CID000007845) is an important industrial chemical used as a monomer in the production of synthetic rubber. Researchers have found that it is a high-efficiency carcinogen [1].

2) Ritonavir (CID000392622) is an antiretroviral drug from the protease inhibitor class used to treat HIV infection and AIDS. It is also a potent inhibitor of CYP3A4, which can enhance the anticancer effects of docetaxel in androgen-independent prostate cancer [2].

3) Swainsonine (CID000051683) is an indolizidine alkaloid, which can activate mitochondria-mediated apoptotic pathway in human lung cancer A549 cells [3].

4) Chitin (CID000024139) is an adjuvant for cancer vaccine [4].

5) Angiotensin II (CID000172197) is a peptide hormone that causes vasoconstriction and a subsequent increase in blood pressure. Research has shown that angiotensin II receptor blockers (ARBs) may be associated with an increased risk for cancer development [5].

6) Colchicine (CID000006167) is an alkaloid agent which has been used in medicine for a long time. Also, it is implied that colchicine is a potential anti-cancer drug for hepatocellular carcinoma [6].

7) Sulfur mustard (CID000010461) is a class of related cytotoxic and vesicant chemical warfare agents with the ability to increase the incidence rates of malignant disorders [7].

8) Methylazoxymethanol (CID006433205) acetate, MAM, is a neurotoxin and carcinogen which reduces DNA synthesis [8].

9) Chloride ion (CID000000312) is a common ion in human cells, which plays a crucial in cell invasion due to its ability to change the osmotic balance between the inner- and extra- cellular space [9]. The reason behind invading cancer cells that can pass though extracellular matrix is partly because it has the ability to reduce its volume. Several major chloride channels on the cell membrane is responsible for this invasive behavior of cancer cells. Research has found that inhibition of the sodium-potassium-chloride co-transporter isoform-1 (NKCC1) can decrease cell invasion by 50% [10].

10) Diclofenac (CID000003032) attenuates Wnt/β-catenin signaling in colon cancer cells by activation of NF-κB [11].

11) EGCG (CID000065064) is a major polyphenols in green tea, which inhibits pancreatic cancer growth [12].

12) Propionate (CID000001032) can reduce cancer cell proliferation in the liver [13].

13) Hydrogen cyanide (HCN, CID000000768) is the product of various tobaccos, existing in the smoke as a colorless gas. In the study of gastro-esophageal cancer based on selected ion flow tube mass spectrometry (SIFT-MS), hydrogen cyanide is significantly different between cancer and healthy groups [14]. Hydrogen cyanide is also recognized to have cardiovascular and respiratory toxicity, which might be a potential factor to cause lung cancer [15].

14) BDCM (CID000006359) can induce cancer in several tissues in experimental animals [16].

15) Citalopram (CID000002771) is an antidepressant drug of the selective serotonin reuptake inhibitor (SSRI) class. Also, it has been proven to induce apoptosis in human acute myeloid leukemia [17].

16) L-leucine (CID000006106) is an essential amino acid, which can induce growth arrest and persistent ERK activation in glioma cells [18].

17) Paroxetine (CID000043815) induces growth inhibition and apoptosis in prostate cancer cells [19].

18) Acetone (CID000000180) is the organic compound with the formula $(CH_3)_2CO$. Acetone extract of Angelica sinensis can inhibit the proliferation of cancer cells in vitro [20].

19) Methylglyoxal (CID000000880) is the organic compound with the formula CH3C(O)CHO, which can induce growth arrest in the G1 phase of the cell cycle and toxicity in human leukaemia 60 cells in vitro [21].

20) Omeprazole (CID000004594) is a proton pump inhibitor, which can inhibit proliferation and modulates autophagy in pancreatic cancer cells [21].

21) Pentoxifylline (CID000004740) is a xanthine derivative, which can induce GSK-3β-independent proteasomal degradation of cyclin D1 and arrest renal cancer cells in the G1 phase [22].

22) The degraded product of phytate (CID000000890) can suppress the proliferation of HCT116 colorectal cancer cells [23].

23) Selenium (CID006326970) is a chemical element with symbol Se and atomic number 34. Selenium supplementation has been found to be a chemopreventive for some types of cancer [24].

24) Malate (CID000000525) plays an important role in biochemistry. Sunitinib malate (SU-11248) alone or in combination with low-dose docetaxel inhibits the growth of DU-145 prostate cancer xenografts [25].

25) Aniline (CID000006115) consists of a phenyl group attached to an amino group, and it is the precursor of industrial chemicals. It is reported that the incidence of bladder cancer is clearly related to exposure to aniline [26]. Potential reasons might be due to an increase in iron overload in the spleen, and up-regulation of TNF-α, IL-1, and IL-6. Also, the expression of cyclin dependant kinases (CDKs) is up-regulated by aniline [27].

26) Monomethylarsonic acid (CID000008947) is the product of arsenic, which shows potential carcinogenic effects [28].

27) D-malate (CID000092824) is suggested to be used to treat cancer [25].

28) Tamoxifen (CID002733525) is an antagonist of the estrogen receptor in breast tissue via its active metabolite, 4-hydroxytamoxifen. Some breast cancer cells require estrogen to grow. Estrogen binds to and activates the estrogen receptor in these cells. Tamoxifen is metabolized into compounds that also bind to the estrogen receptor but do not activate it. Because of this competitive antagonism, tamoxifen acts like a key broken off in the lock that prevents any other key from being inserted, preventing estrogen from binding to its receptor. Hence breast cancer cell growth is blocked [29].

29) Inosine monophosphate (CID000008582) is a nucleoside monophosphate.The up-regulated expression inosine monophosphate dehydrogenase type 2 (IMPDH2) of has been observed in colorectal adenocarcinoma tissues [30].

1. Xiang M, Ao L, Yang H, Liu W, Sun L, et al. (2012) Chromosomal damage and polymorphisms of metabolic genes among 1, 3-butadiene-exposed workers in a matched study in China. Mutagenesis 27: 415-421.
2. Ikezoe T, Hisatake Y, Takeuchi T, Ohtsuki Y, Yang Y, et al. (2004) HIV-1 Protease Inhibitor, Ritonavir A Potent Inhibitor of CYP3A4, Enhanced the Anticancer Effects of Docetaxel in Androgen-Independent Prostate Cancer Cells In vitro and In vivo. Cancer research 64: 7426-7431.
3. Li Z, Xu X, Huang Y, Ding L, Wang Z, et al. (2012) Swainsonine activates mitochondria-mediated apoptotic pathway in human lung cancer A549 cells and retards the growth of lung cancer xenografts. International journal of biological sciences 8: 394.

4. Chen WR (2013) Chitin, chitosan, and glycated chitosan regulate immune responses: the novel adjuvants for cancer vaccine. Clinical and Developmental Immunology 2013.
5. Huang C-C, Chan W-L, Chen Y-C, Chen T-J, Lin S-J, et al. (2011) Angiotensin II receptor blockers and risk of cancer in patients with systemic hypertension. The American journal of cardiology 107: 1028-1033.
6. Lin Z-Y, Wu C-C, Chuang Y-H, Chuang W-L (2013) Anti-cancer mechanisms of clinically acceptable colchicine concentrations on hepatocellular carcinoma. Life sciences 93: 323-328.
7. Zafarghandi MR, Soroush MR, Mahmoodi M, Naieni KH, Ardalan A, et al. (2013) Incidence of cancer in Iranian sulfur mustard exposed veterans: a long-term follow-up cohort study. Cancer Causes & Control 24: 99-105.
8. Hiraku Y, Kawanishi S, Ohshima H (2014) Cancer and Inflammation Mechanisms: Chemical, Biological, and Clinical Aspects: John Wiley & Sons.
9. Veiseh O, Kievit FM, Ellenbogen RG, Zhang M (2011) Cancer cell invasion: treatment and monitoring opportunities in nanomedicine. Advanced drug delivery reviews 63: 582-596.
10. Haas BR, Sontheimer H (2010) Inhibition of the sodium-potassium-chloride cotransporter isoform-1 reduces glioma invasion. Cancer research 70: 5597-5606.
11. Cho M, Gwak J, Park S, Won J, Kim D-E, et al. (2005) Diclofenac attenuates Wnt/β-catenin signaling in colon cancer cells by activation of NF-κB. FEBS letters 579: 4213-4218.
12. Tang S-N, Marsh L, Shankar S, Srivastava RK (2011) EGCG inhibits pancreatic cancer growth and stem cell characteristics in xenografts and KrasG12D transgenic mice models. Cancer Research 71: 859-859.
13. Bindels LB, Porporato P, Dewulf E, Verrax J, Neyrinck AM, et al. (2012) Gut microbiota-derived propionate reduces cancer cell proliferation in the liver. British journal of cancer 107: 1337-1344.
14. Kumar S, Huang J, Cushnir JR, Spanel P, Smith D, et al. (2012) Selected ion flow tube-MS analysis of headspace vapor from gastric content for the diagnosis of gastro-esophageal cancer. Anal Chem 84: 9550-9557.
15. Branton PJ, McAdam KG, Winter DB, Liu C, Duke MG, et al. (2011) Reduction of aldehydes and hydrogen cyanide yields in mainstream cigarette smoke using an amine functionalised ion exchange resin. Chem Cent J 5: 15.
16. Lilly PD, Andersen ME, Ross TM, Pegram RA (1998) A physiologically based pharmacokinetic description of the oral uptake, tissue dosimetry, and rates of metabolism of bromodichloromethane in the male rat. Toxicology and applied pharmacology 150: 205-217.
17. Xia Z, Bergstrand A, DePierre JW, Nässberger L (1999) The antidepressants imipramine, clomipramine, and citalopram induce apoptosis in human acute myeloid leukemia HL-60 cells via caspase-3 activation. Journal of biochemical and molecular toxicology 13: 338-347.
18. Takeuchi S, Nawashiro H, Wada K, Nomura N, Toyooka T, et al. (2012) l-Leucine

induces growth arrest and persistent ERK activation in glioma cells. Amino acids 43: 717-724.

19. Shibuya T (2011) Paroxetine, a selective serotonin re-uptake inhibitor, induces growth inhibition and apoptosis in prostate cancer cells. Cancer Research 71: 2152-2152.
20. Cheng Y-L, Chang W-L, Lee S-C, Liu Y-G, Chen C-J, et al. (2004) Acetone extract of< i> Angelica sinensis</i> inhibits proliferation of human cancer cells via inducing cell cycle arrest and apoptosis. Life sciences 75: 1579-1594.
21. Kang Y, Edwards LG, Thornalley PJ (1996) Effect of methylglyoxal on human leukaemia 60 cell growth: Modification of DNA, G< sub> 1</sub> growth arrest and induction of apoptosis. Leukemia research 20: 397-405.
22. Cai W, Mastrandrea N, Tham K, Monks T, Lau S (2014) Pentoxifylline induces GSK-3β-independent proteasomal degradation of cyclin D1 and arrests renal cancer cells in the G1 phase (616.5). The FASEB Journal 28: 616.615.
23. Ishizuka S, Saitoh K-i, Suzuki T, Lee J-S, Hara H (2011) A partially degraded product of phytate suppresses the proliferation of HCT116 colorectal cancer cells. Food Chemistry 125: 1219-1225.
24. Dennert G, Zwahlen M, Brinkman M, Vinceti M, Zeegers M, et al. (2011) Selenium for preventing cancer. Cochrane Database Syst Rev 5.
25. Cumashi A, Tinari N, Rossi C, Lattanzio R, Natoli C, et al. (2008) Sunitinib malate (SU-11248) alone or in combination with low-dose docetaxel inhibits the growth of DU-145 prostate cancer xenografts. Cancer letters 270: 229-233.
26. Carreón T, Hein M, Hanley K, Viet S, Ruder A (2014) 0094 Bladder cancer incidence among workers exposed to o-toluidine, aniline and nitrobenzene at a rubber chemical manufacturing plant. Occupational and environmental medicine 71: A9-A10.
27. Kannan S, Fielder R, Tristan J, Longoria E, Castillon A (2013) Molecular Mechanism of Aniline induced bladder cancer. The FASEB Journal 27: 793.791.
28. López-Carrillo L, Hernández-Ramírez RU, Gandolfi AJ, Ornelas-Aguirre JM, Torres-Sánchez L, et al. (2014) Arsenic methylation capacity is associated with breast cancer in northern Mexico. Toxicology and applied pharmacology 280: 53-59.
29. Davies C, Pan H, Godwin J, Gray R, Arriagada R, et al. (2013) Long-term effects of continuing adjuvant tamoxifen to 10 years versus stopping at 5 years after diagnosis of oestrogen receptor-positive breast cancer: ATLAS, a randomised trial. The Lancet 381: 805-816.
30. Shim D, Kang JH, Park ES, Kang KR (2010) The expression and characterization of up-regulated inosine monophosphate dehydrogenase type 2 (IMPDH2) in colorectal adenocarcinoma tissues by proteomic analysis. Cancer Research 70: 5558-5558.
